# Green-Synthesized Characterization, Antioxidant and Antibacterial Applications of CtAC/MNPs-Ag Nanocomposites

**DOI:** 10.3390/ph17060772

**Published:** 2024-06-13

**Authors:** Ayşe Baran, Erdal Ertaş, Mehmet Fırat Baran, Aziz Eftekhari, Zübeyir Gunes, Cumali Keskin, Sergey A. Usanov, Rovshan Khalilov

**Affiliations:** 1 Department of Biology, Graduate Education Institute, Mardin Artuklu University, Mardin 47200, Turkey; ayse.gorgec43@gmail.com; 2 Department of Food Technology, Vocational School of Technical Sciences, Batman University, Batman 72000, Turkey; erdalertas21@gmail.com (E.E.); mfiratbaran@gmail.com (M.F.B.); 3 Department of Biochemistry, Faculty of Science, Ege University, Izmir 35040, Turkey; 4 Department of Life Sciences, Western Caspian University, Baku AZ1072, Azerbaijan; 5 Department of Crops and Animal Production, Mardin Artuklu University, Mardin 47200, Turkey; zubeyirgunes@artuklu.edu.tr; 6 Department of Medical Services, Vocational School of Health Services, Mardin Artuklu University, Mardin 47200, Turkey; ckeskinoo@gmail.com; 7 Institute of Bioorganic Chemistry, National Academy of Sciences of Belarus, 220141 Minsk, Belarus; 8 Department of Biophysics and Biochemistry, Baku State University, Baku AZ1148, Azerbaijan; 9 Institute of Radiation Problems, Ministry of Science and Education Republic of Azerbaijan, Baku AZ1143, Azerbaijan

**Keywords:** *Celtis tournefortii*, biological activity, CtAC/MNPs-Ag nanocomposite

## Abstract

The emergence of antibiotic resistance, caused by the improper use of antibiotics, is a significant challenge in combating infectious diseases, leading to millions of annual fatalities. The occurrence of antimicrobial side effects catalyzes the investigation of novel antimicrobial compounds and sources of drugs. Consequently, the research on biological activity that is conducted on plants, plant extracts, and compounds that are produced from plant components is of utmost significance. In this study, CtAC/MNPs were obtained by the reaction of activated carbon (AC) obtained from the fruits of the *Celtis tournefortii* (Ct) plant and magnetic nanoparticles (MNPs), and a CtAC/MNPs-Ag nanocomposite was synthesized by the reduction in silver ions added to the reaction. The synthesized CtAC/MNPs and CtAC/MNPs-Ag nanocomposites were analyzed spectroscopically (FTIR, XRD), microscopically (SEM, EDX), optically (DLS), electrochemically (zeta potential) and magnetically (VSM). The antibacterial activities of CtAC/MNPs and CtAC/MNPs-Ag nanocomposites against *S. aureus* and *E. coli* were investigated by microdilution method using minimal inhibitory concentration (MIC) and disk diffusion methods. Antioxidant activity study, including total phenolic content and DPPH and cuprac assays, revealed the remarkable effect of the CtAC/MNPs-Ag nanocomposite. This study has the advantages of obtaining CtAC/MNPs and CtAC/MNPs-Ag nanocomposites in a short time without requiring energy, and most importantly, the reaction takes place without using any toxic substances. In addition, according to the data obtained in the study, the CtAC/MNPs-Ag nanocomposite is thought to shed light on biomedical research.

## 1. Introduction

Nowadays, new technologies are being researched to reduce the size of materials and lighten them. This has occurred as a consequence of the application of nanotechnology, which enhances the properties of materials [1]. Nanotechnology is responsible for the development and production of the required nanoparticles [2,3]. Nanoparticles are materials that range in size from one to one hundred nanometers. These small particles have been utilized in numerous applications [4,5]

To comprehend and utilize nanoparticles, it is necessary to categorize them based on their size, shape, and content [6,7,8]. Nanoparticles are commonly classified based on their size. Nanoparticles are categorized based on their size into three groups: ultrafine (1–100 nm), fine (100–2500 nm), and coarse (>2500 nm) [9]. The size classification of nanoparticles determines their surface area, reactivity, and toxicity [10]. Morphology is another method of nanoparticle classification. Nanoparticles can exhibit several morphologies, including spherical, rod-like, tubular, or wire-like structures [11,12]. The shape of the nanoparticle influences its optical, electrical, and mechanical characteristics. Furthermore, it can be classified according to the nanoparticle composition. The chemical stability, biocompatibility, and magnetic properties of nanoparticles vary depending on the component [13].

Due to their unique properties, the potential applications of metal or metallic nanoparticles in various fields such as medicine, electronics, and environmental science have been the subject of intense research and interest in recent years. These nanoparticles consist of metal atoms typically less than 100 nm, giving them a large surface area to volume ratio and distinctive physicochemical properties. Metal nanoparticles have become one of the most important components of nanotechnology, which has attracted great attention in many fields in recent years. These nanoparticles are generally obtained from metals such as gold, copper, silver, titanium, palladium, and platinum [14,15].

Silver nanoparticles are an important nanotechnology product that has been widely used in many industrial and medical fields in recent years. Silver nanoparticles are preferred in many applications due to their small size and unique properties [16,17]. Due to their small size, silver nanoparticles can be easily applied to the surface of many materials and positively affect the properties of the material. Additionally, silver nanoparticles have become one the popular study subjects due to their wide range of applications such as environmental friendliness, pollution reduction, energy saving, electrical and thermal conductivity, anti-cancer, anti-inflammatory, drug transport, wound healing, biosensor, antimicrobial, antioxidant and biocatalysts [18,19].

Activated carbon, also known as activated charcoal, is a highly porous material widely used for water purification, air filtration, and removal of impurities in various substances. One of the sources of activated carbon is plants. The process of obtaining activated carbon from plant sources involves heating the raw material at high temperatures in the presence of steam. This creates small pores and cracks in the carbon structure, increasing its surface area and resulting in an increase in surface capacity. Activated carbon can then be processed into various forms such as powder, granules, or pellets, depending on the intended application. Production of activated carbon using the fruit part of plants is a sustainable and environmentally friendly method of obtaining this valuable material [20,21,22].

Support materials are used to increase the activity and selectivity of silver nanoparticles, but activated carbon has proven to be one of the most effective options for this purpose [23]. The success of activated carbon in supporting nanoparticles increases the thermal and mechanical stability of the material and maximizes catalytic efficiency by increasing the effective surface area thanks to its high surface area [24]. However, since activated carbon is biologically compatible with microorganisms and can increase the proliferation of bacteria, it would be advantageous to use silver to give activated carbon antibacterial properties to eliminate this disadvantage that may occur during its use in different applications [25]. In addition, by binding silver ions to a support material to produce silver-bonded (doped) nanocomposites, agglomeration of nanocomposites can be prevented, and recycling and reuse of silver nanoparticles supported on activated carbon in various industrial applications can be achieved [26]. Moreover, activated carbon stands out as an ideal support material for silver nanoparticles due to its high surface area and pore volume [27].

MNP nanoparticles are important materials widely used in many industrial and medical fields today. The modification of these nanoparticles is especially important for biomedical applications because the control of surface properties is important for effectively directing the nanoparticles to targeted cells. Activated carbon obtained from Dardagan fruit is a potential material for surface modification of nanoparticles. The effect of activated carbon on MNPs may offer advantages such as increasing the biocompatibility of nanoparticles, improving their target orientation, and enabling their more effective use in environmental applications. The use of activated carbon obtained from daffodil fruit for the modification of MNPS may offer significant advantages to enable the more effective use of these nanoparticles in biological and environmental applications [28,29,30].

Dardagan, also known by its scientific name *Celtis tournefortii*, is a species of flowering plant belonging to the Cannabaceae family. Native to the Middle East, particularly Iran and Iraq, the Dardagan is a small to medium-sized species that can reach heights of up to 15 m. It is known for its distinctive appearance, with dark green, oval leaves and small, round fruits that turn purplish-black when ripe [31,32].

One of the most striking features of Dardagan is its adaptability to various climates and soil types. It can grow in both arid and semi-arid regions and is also a plant species that is resistant to drought and requires low maintenance.

In this study, for the first time, the antimicrobial activity effects of CtAC/MNPs and CtAC/MNPs-Ag nanocomposites obtained by using the Ct plant on *E. coli* and *S. aureus* bacteria and the determination of the antioxidant activity effects and total phenolic content of the CtAC/MNPs-Ag nanocomposite are comprehensively explained.

## 2. Result and Discussion

### 2.1. FT-IR Analysis

**Figure 1:**
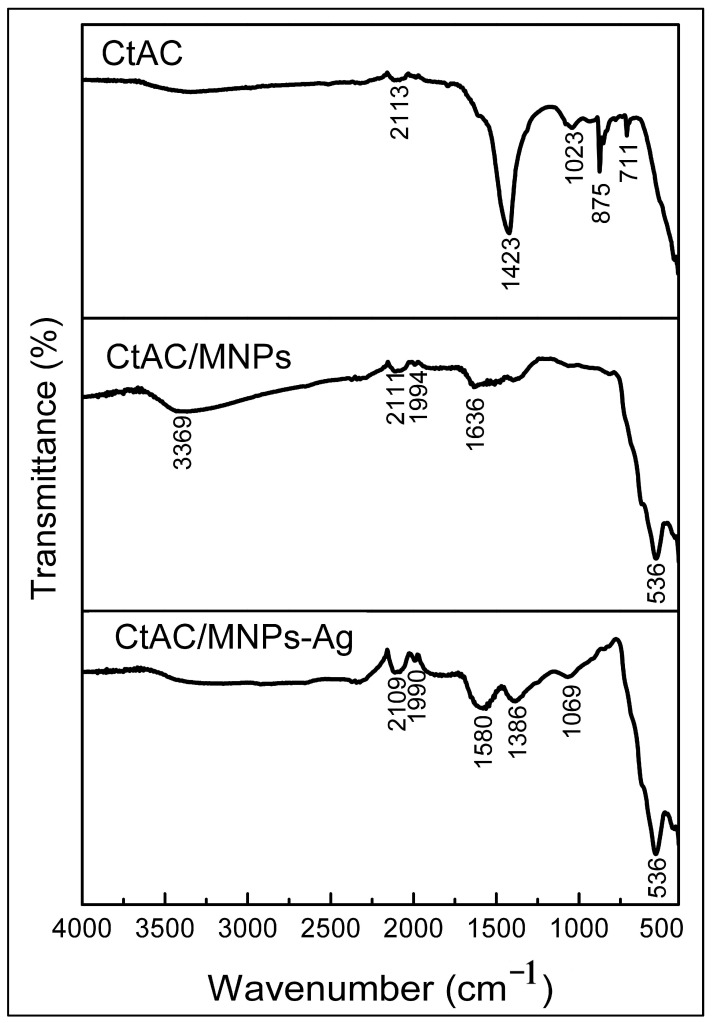
FTIR spectrum comparison of CtAC, CtAC/MNPs, and CtAC/MNPs-Ag.

*Celtis tournefortii* fruits were used as biological sources in the synthesis of both AC-MNPs and Ag nanoparticles. The only source of phenolic compounds in the synthesized CtAC/MNPs-Ag nanocomposite was the extract obtained from *Celtis tournefortii* fruits. Spectrum shifts detected in the FTIR spectrum indicate the presence of functional groups of the compounds in the extract. FTIR spectroscopy was used to evaluate the synthesis of CtAC composite, CtAC/MNPs, and CtAC/MNPs-Ag nanocomposites. The results are presented in Figure 1. The CtAC composite’s FT-IR spectrum has a peak at 2113 cm^−1^, which corresponds to the C=C stretching vibration of alkyne [33,34]. In addition, the peaks observed at 1423 and 1023 cm^−1^ were attributed to the stretching vibrations of -CH_2_ and C−OH [35] while the peaks at 875 and 711 cm^−1^ are probably attributable to out-of-plane deformation of C=C and C-H bonds in the benzene ring [35,36].

The 3369 cm^−1^ peak of the CtAC/MNPs nanocomposite can be assigned to the O-H bond due to the hydroxyl on the surface of the structure. hydroxyl groups (OH), possibly attributable to the presence of water molecules that do not fully dry on the surface of the nanocomposite for analysis after synthesis, the formation of long-chain acid and hydroxy compounds, or the decomposition or recombination of oxygen-containing rings [37,38,39]. The analysis of the spectra of CtAC/MNPs and CtAC/MNPs-Ag nanocomposites reveals the existence of alkyne and C-H groups, as evidenced by the absorbance peaks observed at 2109, 2113, 2011, 1994, and 1990 cm^−1^. The observed peaks at 1636 and 1580 cm^−1^, which correspond to the carbonyl C=O group. The increase in the peak intensity of C=O groups, which was not visible in the FTIR analysis of CtAC, indicates that the functional groups on the surface of the activated carbon structure improve the peak intensity as a result of surface modification of the activated carbon with MNPs [40]. Furthermore, the absorption peak observed at 1386 cm^−1^ in CtAC/MNPs and CtAC/MNPs-Ag nanocomposites signifies the bending vibration of –OH and C-H bonds, whereas the peak at 1069 cm^−1^ indicates the existence of C-O and C-N functional groups [41]. The strong peak observed at 536 cm^−1^ in Figure 1 is related to the presence of the Fe-O-Fe stretching vibration component in the core of CtAC/MNPs and CtAC/MNPs-Ag nanocomposites [42]. FT-IR results show that Fe_3_O_4_ is indeed dispersed with various functional bonds (such as hydroxyl, carbonyl, and amine groups) abundantly attached to the porous surfaces of activated carbon [35].

### 2.2. SEM and EDX Analysis

**Figure 2:**
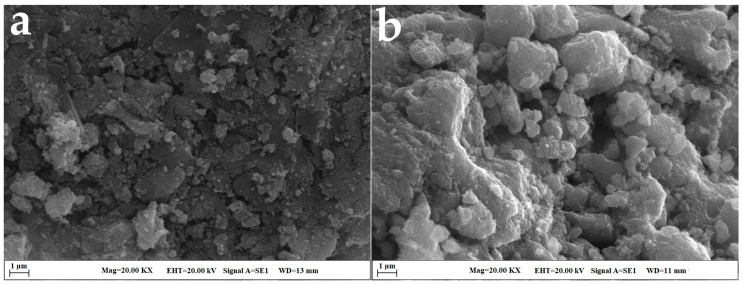
SEM images (**a**) CtAC/MNPs; (**b**) CtAC/MNPs-Ag.

**Figure 3:**
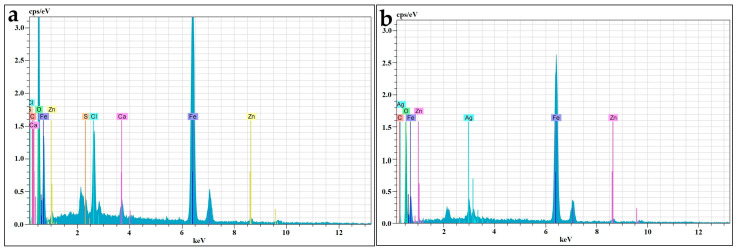
EDX images (**a**) CtAC/MNPs; (**b**) CtAC/MNPs-Ag.

The surface morphologies of CtAC/MNPs and CtAC/MNPs-Ag nanocomposites were examined using scanning electron microscopy (SEM). The elemental composition of these nanocomposites was determined by EDX analysis. This study presents scanning electron microscopy (SEM) images of CtAC/MNPs and CtAC/MNPs-Ag nanocomposites in Figure 2a,b, while EDX spectra are depicted in Figure 3a,b.

The nanocomposite of CtAC/MNPs exhibits a granular surface with diverse pore sizes. The surface form and pore structures of the CtAC/MNPs-Ag nanocomposite undergo modifications after its acquisition. The presence of heterogeneity and irregular distribution on the surface of the CtAC/MNPs composite leads to the attachment and subsequent smoothing of Ag ions [43,44].

The EDX analysis of the CtAC/MNPs nanocomposite indicates the presence of carbon (C), oxygen (O), zinc (Zn), and iron (Fe), which are important components of the nanocomposite. In the process of analyzing the CtAC/MNPs-Ag nanocomposite using EDX, it was observed that the element silver (Ag) was also detected. The relative amounts of Fe and Ag in CtAC/MNPs-Ag were measured as 53% and 2%, respectively. Iron and silver components have been verified in CtAC/MNPs and CtAC/MNPs-Ag nanocomposites using EDX analysis, as depicted in Figure 3a,b [45,46,47].

### 2.3. Particle Size and Zeta Potential Analysis

**Figure 4:**
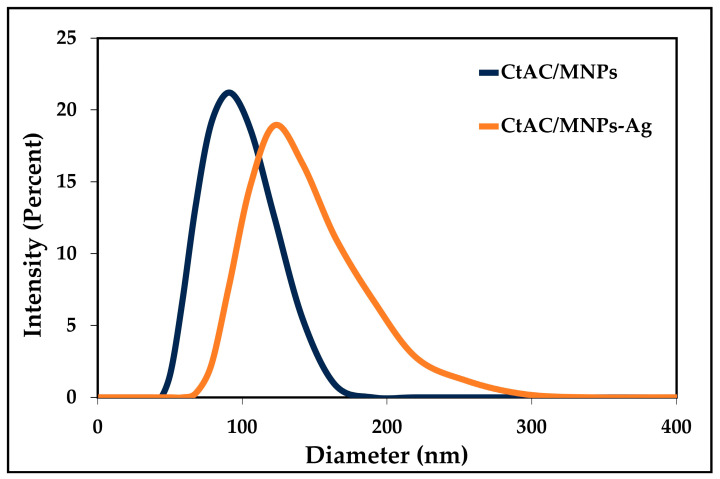
DLS analysis of CtAC/MNPs and CtAC/MNPs-Ag.

In Figure 4, the results of the DLS analysis are presented following the dispersion of 10 mg of CtAC/MNPs and CtAC/MNPs-Ag nanocomposites in 100 mL of purified water using an ultrasonic bath. The particle size distribution inside a solid–liquid mixture can be determined through the utilization of DLS analysis. As an illustration, upon examination of the outcomes derived from the DLS analysis, it is evident that the mean size distribution of CtAC/MNPs is 91 nm, while the mean size distribution of CtAC/MNPs-Ag is 122 nm [48].

**Figure 5:**
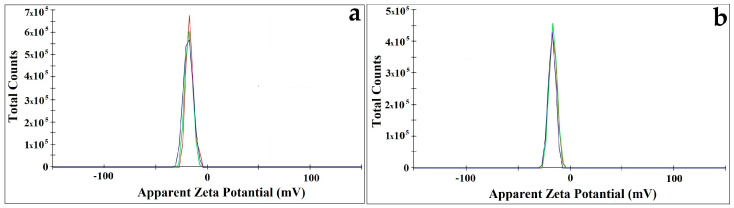
Zeta potential for (**a**) CtAC/MNPs (**b**) CtAC/MNPs-Ag nanocomposite.

The zeta potential study of CtAC/MNPs and CtAC/MNPs-Ag nanocomposites is shown in Figure 5. The zeta potential on any material gives its surface electric charge. To prevent agglomeration and clustering of CtAC/MNPs and CtAC/MNPs-Ag nanocomposites and assure stability, high positive or negative zeta potential findings are needed. Under optimal conditions, CtAC/MNPs and CtAC/MNPs-Ag nanocomposites had potential values of −17.0 and −16.6 mV. In line with this, CtAC/MNPs and CtAC/MNPs-Ag nanocomposites had little agglomeration and stable suspensions for months [49,50,51].

### 2.4. VSM Analysis

**Figure 6:**
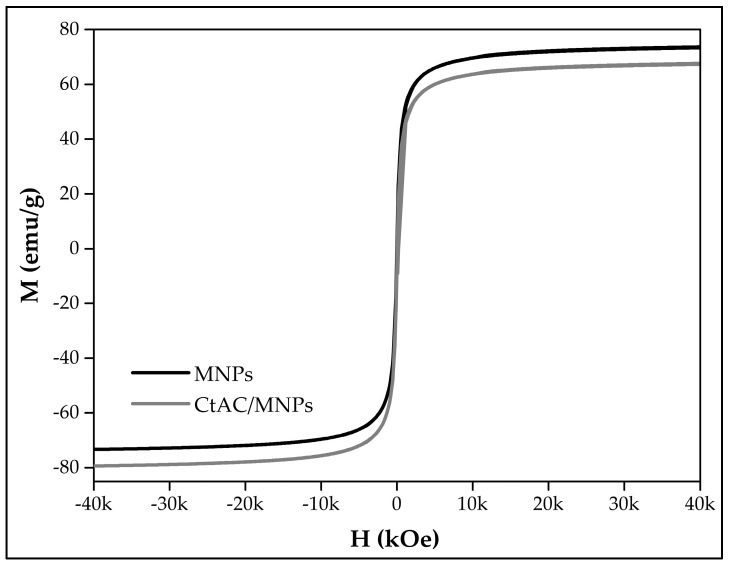
VSM analysis of MNPs and CtAC/MNPs nanocomposite.

Figure 6 shows the MNPs’s and CtAC/MNPs nanocomposite’s magnetic behavior. The magnetic saturation value for MNPs was 73.7 emu/g, and for the CtAC/MNPs nanocomposite, it was 67.72 emu/g. The magnetic saturation parameter of the CtAC/MNPs nanocomposite might vary depending on particle size, concentration, and oxygen absorption [36].

### 2.5. XRD Analysis CtAC/MNPs and CtAC/MNPs-Ag Nanocomposites

**Figure 7:**
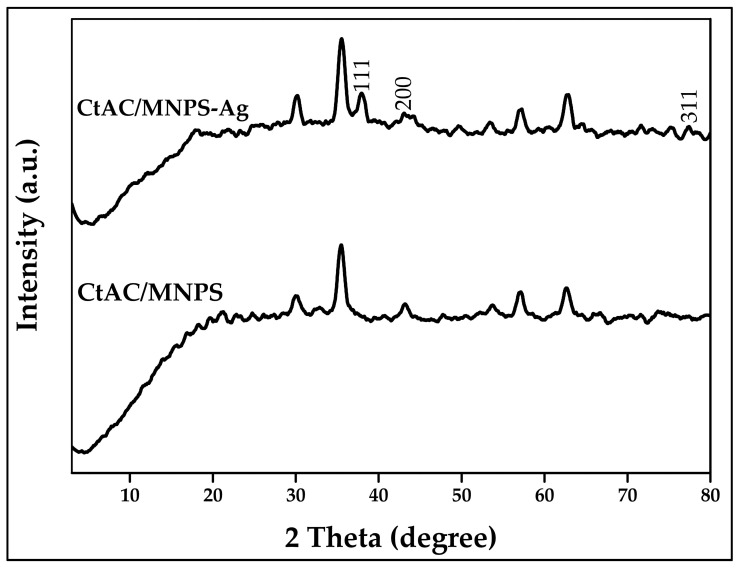
XRD patterns of CtAC/MNPs and CtAC/MNPs-Ag.

Figure 7 displays the X-ray diffraction pattern of CtAC/MNPs and CtAC/MNPs-Ag nanocomposites’ crystal phases. The CtAC/MNPs nanocomposite exhibits crystal reflection patterns of (111) and (200), whereas the CtAC/MNPs-Ag nanocomposite displays crystal reflection patterns of (111), (200) and (311) [52]. In this work, the Joint Committee on Powder Diffraction Standards (JCPDS) value of the CtAC/MNPs nanocomposite was 28-0491, and for CtAC/MNPs-Ag nanocomposites, it was 65-2871 [53]. According to the Scherrer equation, the greatest peak values of 35.440 and 38.021 determined the crystal size of CtAC/MNPs and CtAC/MNPs-Ag nanocomposites as 24.67 and 28.59 nm. XRD shows that CtAC/MNPs and CtAC/MNPs-Ag nanocomposites were synthesized.

The Ct-ACMnNPs and Ct-ACMnNPs crystal dimensions were determined using the D = Kλ/(β cosθ) formula. D is the particle size, K is the constant value (1.504), λ is the X-ray wavelength value (0.90), β is half of the FWHM value of the peak with the maximum height, and θ is the high peak’s Bragg angle (Ct-ACMNPs = 0.591 Ct-ACMNPs-Ag = 0.513) in the formula.

### 2.6. CtAC/MNPs and CtAC/MNPs-Ag Nanocomposites Antibacterial Effect

Disc diffusion and microdilution methods were employed to study the antibacterial properties of the CtAC/MNPs-Ag and CtAC/MNPs nanocomposite. The data from Table 1 show that the CtAC/MNPs-Ag nanocomposite had a substantial impact on two different bacterial species: Gram-positive and Gram-negative. The CtAC/MNPs-Ag nanocomposite demonstrated greater efficacy against Gram-positive *S. aureus* in the microdilution test, requiring a lower dosage than the antibiotic. This result is additionally corroborated by the disc diffusion approach (Table 1). The CtAC/MNPs-Ag nanocomposite exhibited higher efficacy against *S. aureus* bacteria compared to *E. coli*. Gram-positive bacteria possess a more substantial peptidoglycan coating compared to Gram-negative bacteria. Within this stratum, linear polysaccharide chains are connected by cross-links containing short peptides. Conversely, the wall architectures of Gram-negative bacteria are intricate. Gram-negative bacteria possess lipopolysaccharide plates, a feature absent in Gram-positive bacteria. The variation in wall architectures significantly influences how nano-sized objects impact two distinct species at varying concentrations [54,55,56].

**Figure 8:**
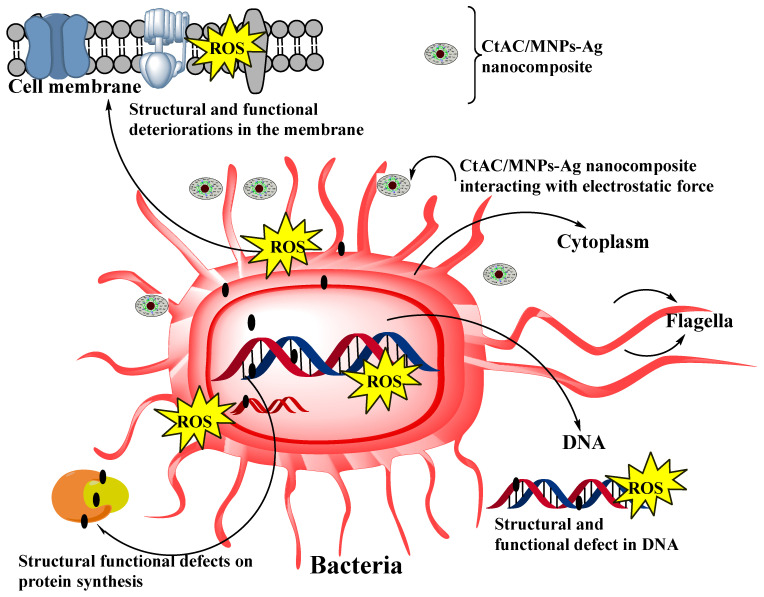
Mechanism of antimicrobial activity.

Metallic nanoparticles are valuable products that are constantly being developed due to their multiple antimicrobial effects as opposed to the specific effect of the antibiotic [57,58,59]. The antibacterial properties of silver ions have long been recognized. Silver ions that are ionized or diffuse exhibit high reactivity. Through the electrostatic force of attraction, they come together and interact with other microbes in the same environment. The effects that are shown in Figure 8 are assumed to be the mechanism that is responsible for the inactivation of bacteria by CtAC/MNPs-Ag. Several reports have suggested that this inactivation is related to the production of reactive oxygen species (ROS), depletion of antioxidants, and protein dysfunction (Figure 8) [60,61,62,63]. Considering the aforementioned works and the discoveries of the current investigation, we have put up a credible and comprehensive procedure. The antimicrobial action may occur in two different aspects. Ag depletion may lead to the creation of irregularly shaped pits in the outer bacterial membrane and alter its permeability, resulting in the leaking of cellular substances. Alternatively, we hypothesized that the antibacterial action of CtAC/MNPs-Ag was linked to the generation of free radicals, which in turn led to membrane degradation. Then, because of the rise in reactive oxygen species (ROS), they result in flaws in the membrane structure of microorganisms. Due to its strong affinity for reactive oxygen species (ROS), RNA disrupts the structure and activities of essential biomolecules like DNA and essential enzymes. Microorganisms perish all of these factors that obstruct essential functions (Figure 8) [56,60,61,62,63,64,65,66].

### 2.7. The Total Phenolic Content

The total phenolic content was evaluated by creating a calibration curve using five different amounts of gallic acid. The plant extract gallic acid equivalents (mg GAE/g) were calculated based on this curve. The total phenolic content of 1 mg/mL aqueous extracts of nanoparticles derived from plant extracts is presented in Table 2. The morphology of the CtAC/MNPs-Ag nanocomposite remains unchanged; however, the phenolic content of the plant extract has a substantial impact on the phenolic content of the nanoparticles. The overall phenolic content of the CtAC/MNPs-Ag nanocomposite was found to be 10.68 ± 0.435 mg GAE/g, as reported in reference [67].

### 2.8. Antioxidant Capacity

DPPH free radical scavenging experiments compared the CtAC/MNPs-Ag nanocomposite to crude extracts for antioxidant activity. Hydrogen ions and antioxidant compound free electrons neutralize the nitrogen atom’s unpaired electron in the DPPH assay. This turns purple DPPH into yellow hydrazine. The CtAC/MNPs-Ag nanocomposite was evaluated for antioxidant activity by the DPPH radical scavenging method (Table 2). The CtAC/MNPs-Ag nanocomposite at the highest concentration showed an inhibition percentage of 70.37 ± 0.64. The spherical CtAC/MNPs-Ag nanocomposite may have better antioxidant capabilities than the extract due to bioactive component adsorption. Polyphenols’ antioxidant properties and the CtAC/MNPs-Ag nanocomposite’s catalytic actions may also help [68]. The CtAC/MNPs-Ag nanocomposite, produced using nanoparticles, showed a Copper Ion Reducing Antioxidant Capacity (CUPRAC) of 32.70 ± 1.035 (Table 2).

### 2.9. Comparison of Antimicrobial Effect of Various Silver Nanoparticles

The diameter of the inhibition zones (in millimeters) of silver nanoparticles synthesized from different structures against *S. aureus* and *E. coli* using the disc diffusion method is shown in Table 3.

## 3. Materials and Methods

### 3.1. Chemicals and Reagents

All chemicals were used without any further purification. Ferric chloride hexahydrate (FeCl_3_·6H_2_O, ≥99% purity), ferrous chloride tetrahydrate (FeCl_2_·4H_2_O, 98% purity), silver nitrate (AgNO_3_ ≥ 99.0% purity), sodium borohydride (NaBH_4_, 99% purity), hydrochloric acid (HCl, 37% purity), sodium hydroxide (NaOH ≥ 97.0% purity), ammonium hydroxide (NH_3_, 28.0–30.0% purity), and zinc chloride (ZnCl_2_, ≥98% purity) purchased from Sigma-Aldrich (St. Louis, MO, USA). All chemicals were used without any further purification. In this study, deionized (DI) water was used in the synthesis phase, during solution preparation, and for other purposes.

### 3.2. Synthesis of CtAC

In the process of synthesizing activated carbon, the fruit parts of the Ct plant were subjected to initial washing and drying steps to eliminate foreign contaminants. The fruits were pulverized to increase the activation surface area. A quantity of 10 g of powdered plant material was measured and placed into a 500 mL conical flask. Then, a solution of zinc chloride with a concentration of 1 M (during the activation process, the use of activating agents such as ZnCl_2_ tends to promote dehydration of activated carbon and can lead to charring and aromatization with the formation of pores) [35] was added and mixed. The mixture was then subjected to stirring in a water bath set at a temperature of 90 °C for one hour.

Following cooling to ambient temperature, the mixed precipitate was homogeneously put in a big container and dried in an oven at 110 °C for 24 h. Then, the dried mixture was transferred to porcelain crucibles under room conditions and carbonized in a muffle furnace at 550 °C for 4 h, and thus, activated carbon was synthesized (Ct-AC) using *Celtis tournefourtii* fruits (Dardagan). Carbonization increases the surface area by creating pores in the carbon structure. The HCl solution was washed several times with 0.1 N to remove the remaining ions that did not react on the surface of CtAC. After washing the HCl solution, the composite was washed with distilled water and left to dry in an oven set at 75 °C [35,76].

### 3.3. Synthesis of CtAC/MNPs Nanocomposite

CtAC/MNPs were synthesized by the co-precipitation method, which is a simple and useful approach, with some modifications as stated in the literature [77]. After 6.0 g of iron (III) hexahydrate was dissolved in 100 mL of distilled water, a few drops of concentrated HCl solution were added to the solution to avoid hydrolysis of iron (III) ions in the solution environment. In the next step, 4 g of iron (II) tetrahydrate was added to the solution and the temperature of the solution mixture was slowly increased to 90 °C this process continued for 30 min and the solution was mixed. At the end of 30 min, 10 mL of 28% ammonia solution was added to the mixture and stirring was continued for another 30 min at this temperature until the solution turned black. In the next step, the prepared solution of 0.50 g of CtAC in 50 mL of water was added to the mixture. One hour after the addition of CtAC, the system was turned off in a shaking water bath and the CtAC/MNPs obtained were allowed to cool at room temperature. The synthesized CtAC/MNPs nanocomposite was separated from the mixture with an external magnet, washed, and dried in a desiccator at room temperature [43,78]. To remove the substances that did not react with CtAC/MNPs and remained in the environment, the CtAC/MNPs nanocomposite was washed 4–5 times with distilled water and then left to dry in the oven (set at 75 °C). The CtAC/MNPs nanocomposite was stored in a dark environment for use in other studies.

### 3.4. Synthesis of CtAC/MNPs-Ag Nanocomposite

**Figure 9:**
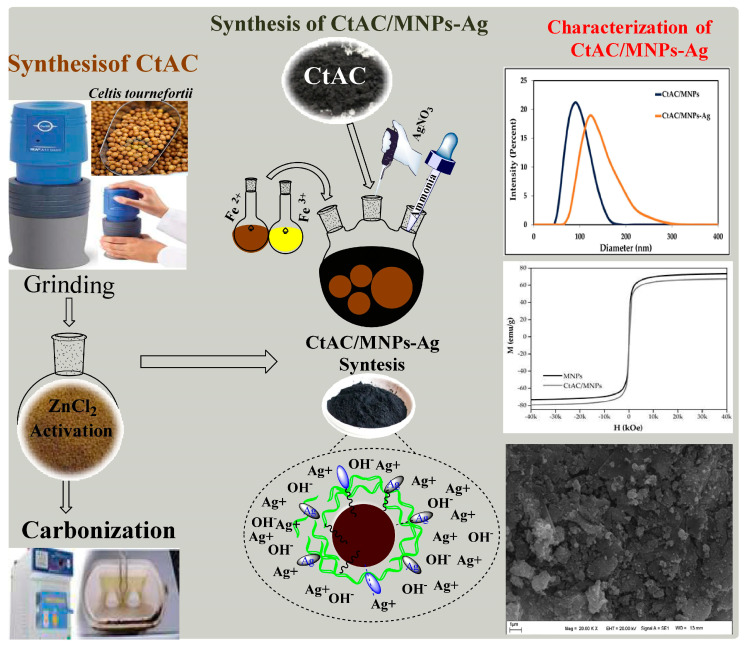
Synthesis mechanism of the CtAC/MNPS-Ag nanocomposite.

For the synthesis of the CtAC/MNPs-Ag nanocomposite, 50 mL of deionized water was added to 1 g of the CtAC/MNPs nanocomposite and dispersed in the ultrasonic bath. After 25 mL of 5 mM AgNO_3_ solution was slowly added to the mixture, mixing was continued for 1 h at 70 °C in the magnetic stirrer. In the next stage, to reduce the silver ions in the mixture to Ag-containing nanoparticles, 0.5 g of NaBH_4_ was quickly added to the mixed solution, and the solution at 75 °C was shaken vigorously in the mixer for 3 h. The synthesized CtAC/MNPs-Ag nanocomposite was removed from the solution environment by an external magnet [44,79,80]. The synthesis mechanism of the CtAC/MNPS-Ag nanocomposite is shown in Figure 9.

### 3.5. Characterization

The FTIR spectra of CtAC, CtAC/MNPs, and CtAC/MNPs-Ag nanocomposites were determined with an Agilent Cary 630 FTIR-ATR spectrometer at a wavelength range of 400–4000 cm^−1^. XRD patterns for the CtAC/MNPs and CtAC/MNPs-Ag nanocomposites were performed using a Rigaku RadB- Dmax II at 2θ = 3°–80° with a scan rate of 1°/min. Surface images of CtAC/MNPs and CtAC/MNPs-Ag nanocomposites were taken using a LEO-EVO 40/Cambridge electron microscope at an accelerating voltage of 20 kV, and a 125 eV Bruker detector EDX system mounted on this system was used. The magnetic properties of CtAC/MNPs and CtAC/MNPs-Ag nanocomposites were observed at 25 °C using a VSM (Quantum Design PPMS-9T). Particle size and zeta potential of CtAC/MNP and CtAC/MNPs-Ag nanocomposites were measured using Zetasizer Nano ZS90 (Malvern Co., Malvern, UK). Before measurements were taken, the samples were used from the stock solution by making the necessary dilutions with pure water.

### 3.6. CtAC/MNPs-Ag and CtAC/MNPs Nanocomposites Antibacterial Effect

The antibacterial properties of CtAC/MNPs-Ag and CtAC/MNPs nanocomposites on the pathogens *E. coli* ATCC 25922 and *S. aureus* ATCC 25923 were investigated by the Disc Diffusion (1) and Micro Dilution (2) methods. The bacteria were cultured in the suitable media under optimal conditions in an incubator set at 37 °C. Merck’s Mueller-Hinton agar and broth were utilized to assess the antibacterial impact. Microorganism strains were produced with a concentration of 1.5 × 10^8^ CFU/mL using the McFarland 0.5 turbidity standard [81,82].

After loading sterile discs with CtAC/MNPs-Ag and CtAC/MNPs nanocomposites, as well as a control with sterile distilled water, each microbe was grown on a Muller–Hinton agar medium. The discs were placed in Petri dishes and incubated at 37 °C for 24 h to allow the active components to interact with the dish. After the period ended, observations were conducted, and the inhibition zones were measured in millimeters.

Various concentrations of CtAC/MNPs-Ag and CtAC/MNP nanocomposites were diluted using a micropipette and added to the wells of a microplate serially. The antibiotics underwent the same microdilution processes for comparison. Bacterial suspensions made using the McFarland 0.5 turbidity standard were added to each well, and the microplates were incubated at 37 degrees Celsius for 24 h. After the experiment, a growth control was conducted to establish the Minimum Inhibitory Concentrations (MIC) at which growth was inhibited.

### 3.7. Determination of Total Phenol Content

The phenolic substance of the CtAC/MNPs-Ag nanocomposite was selected using a Folin–Ciocalteu reagent, which may consist of phosphotungstate phosphomolybdate, and gallic acid (as a standard). The phenolic compounds were observed to undergo complexation with the Folin–Ciocalteu reagent under standard circumstances, leading to the formation of a predominantly pure purple-violet color. Following a 2 h incubation period, the maximum absorbance of these compounds was obtained at a wavelength of 760 nm. The analysis results were computed in terms of gallic acid equivalent (mg GAE/g) [83].

### 3.8. Determination of the Efficacy of CtAC/MNPs-Ag Nanocomposite to Scavenge DPPH Radicals

The DPPH (1,1-diphenyl-2-picrylhydrazyl) assay was employed to assess the antioxidant potential of the CtAC/MNPs-Ag nanocomposite in scavenging free radicals. Initially, a solution of DPPH reagent at a concentration of 0.1 mM was produced using methanol. Next, a mixture was prepared by combining 0.4 mL of a 1000 μg/mL CtAC/MNPs-Ag nanocomposite with 0.4 mL of a 0.1 mM DPPH reagent solution, along with 3.2 mL of methanol. As the standard reference, ascorbic acid was employed, and the DPPH scavenging activity was assessed at various particle concentrations (50, 100, and 200 µg/mL). After a comprehensive mixing process, the mixtures were allowed to incubate in a light-free environment at ambient temperature for a duration of 30 min [84]. Subsequently, absorbance measurements were conducted at a wavelength of 517 nm, and the percentage of DPPH radical scavenging was determined utilizing the specified mathematical procedure.

$$\mathrm{Scavenging}\;\mathrm{effect}\;\left(\%\right)=\frac{{(A}_{\mathrm{control}}{-A}_{\mathrm{sample}})}{A_{\mathrm{control}}}\times 100$$

In this context, A_control_ denotes the absorbance of the reaction mixture in the absence of samples, while A_sample_ signifies the absorbance of the reaction mixture when samples are present. The DPPH radical percentage scavenging was determined using the average values obtained from three replicates [85].

### 3.9. Determination of Reduction Force According to the Cuprac Method

The CUPRAC assay for the produced CtAC/MNPs-Ag nanocomposite was modified by Apak et al. (2008) [86]. Test tubes included 1 mL of a CMAC/MNPs-Ag bio nanocomposite and standard antioxidant solutions at 100–1000 μg/mL level. After that, 1 mL of 10 mM Copper (II) Chloride), 7.5 mM neocuprin in ethanol, and 1 mL of 1 M NH_4_Ac buffer solution (pH: 7) were added to the test tubes, followed by distilled water and the final solution. After vortexing, the 8 mL mixture was incubated at room temperature for 30 min. The combination absorbed 450 nm.

## 4. Conclusions

Activated carbon material with a high surface area was synthesized from the fruit of the Ct plant by chemical activation of ZnCl_2_. The CtAC/MNPs nanocomposite was formed by combining activated carbon derived from the Ct plant with magnetic nanoparticles produced through co-precipitation. Then, silver ions were effectively doped into ACs by chemical means, using sodium borohydride as the reducing agent. Nanocomposites characterized by SEM, XRD, FTIR, VSM, DLS, and zeta potential techniques show the formation of activated carbon and silver nanoparticles. In the XRD analysis, the crystal structure of CtAC/MNPs and CtAC/MNPs-Ag nanocomposites were examined and their dimensions were measured as 24.80 and 28.59 nm, respectively. It was determined that the zeta potentials of CtAC/MNPs and CtAC/MNPs-Ag nanocomposites had values of −17.0 and −16.6 mV. Moreover, the PDI average values of CtAC/MNPs and CtAC/MNPs-Ag nanocomposites were found to be 0.701 ± 0.186 and 0.546 ± 0.112, respectively. The total phenolic substance content of the CtAC-MNPs-Ag nanocomposite was determined as 10.68 ± 0.435 mg GAE/g, the DPPH radical scavenging activity was determined as %70.37 ± 0.64, and the CUPRAC reducing power activity was determined as 32.70 ± 1.035 μM TE/100 mL. The antibacterial activity of CtAC/MNPs and CtAC/MNPs-Ag nanocomposites against *E. coli* and *S. aureus* is shown in Table 1. CMAC-MNPs nanocomposites shows higher antibacterial activity than the CtAC/MNPs-Ag nanocomposite. CtAC/MNPs and CtAC/MNPs-Ag nanocomposites can potentially be used as antibacterial agents in various products.

## Figures and Tables

**Table 1:** Gram (−) *E. coli* and gram (+) *S. aureus* bacteria were treated with colistin and vancomycin antibiotics, respectively, for comparative analysis. DW: Distilled water.

MICROORGANISM	MICRODILUTION METHOD (µg/mL)			DISC DIFFUSION METHOD Inhibition Zone (mm)				
	Antibiotic	CtAC/MNPs	CtAC/MNPs-Ag	Control Disc		CtAC/MNPs-Ag	Image	
				DW	CtAC/MNPs		CtAC/MNPs	CtAC/MNPs-Ag
*S. aureus*	1.00	32	1.17	1.00	0.00	12.00	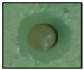	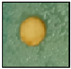
*E. coli*	2.00	128	2.34	2.00	0.00	11.00	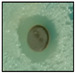	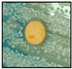

**Table 2:** Total phenolic content and antioxidant capacity (DPPH and CUPRAC) of CtAC/MNPs-Ag nanocomposite.

Samples	Total Phenolic Content (mg GAE/g)	DPPH (%)	CUPRAC (μM TE/100 mL)
CtAC/MNPs-Ag	10.68 ± 0.435	70.37 ± 0.64	32.70 ± 1.035

**Table 3:** A comparison of antimicrobial effect of different silver nanoparticles for *S. aureus* and *E. coli*.

Samples	*S.aureus*	*E. coli*	References
AgNP (*Ferocactus echidne*)	2.10	1.80	[69]
AgNP (*Ziziphus spina-christi*)	5.20	4.40	[70]
AgNP (*Streptomyces enissocaesilis*)	16.60	16.30	[71]
AgNP (*Duchesnea ındica*)	11.64	11.35	[72]
Ag/CNSAC	11.00	8.00	[73]
AgNP (*Linumusitatissimum* L.)	8.90	11.00	[74]
AgNP (*Zanthoxylum armatum*)	21.00	19.00	[75]
**CtAC/MNPs-Ag**	**12.00**	**11.00**	**This study**

## Data Availability

All data and materials are available.
